# Untargeted metabolomics-based response analysis of temperature and insecticide exposure in *Aedes aegypti*

**DOI:** 10.1038/s41598-022-05630-z

**Published:** 2022-02-08

**Authors:** Poonam Singh, Pradeep Kumar, Veena Pande, Virendra Kumar, Ramesh C. Dhiman

**Affiliations:** 1 ICMR National Institute of Malaria Research, New Delhi, Delhi India; 2 Department of NMR and MRI Facility, All India Institute of Medical Sciences, New Delhi, India; 3 Department of Biotechnology, Kumaun University, Nainital, India

**Keywords:** Biological techniques, Molecular biology, Physiology

## Abstract

In this study, we utilized an untargeted NMR metabolomics approach to identify the vector response in terms of metabolic profiling after temperature and insecticide exposure in comparison with the control. Clearly, temperature and insecticide exposure cause changes in the underlying metabolism, and the NMR metabolomic profile enables a direct examination of the immediate response of the vector to cope up with these changes. The present study was designed in four parts: A-*Aedes aegypti* were exposed to 40 °C for one-hour, DDT-4%, malathion-5%, and deltamethrin-0.05% separately and, part B-D; one-hour exposure at 35 °C and 40 °C temperatures followed by one-hour exposure to insecticide. The resultant metabolite profiles were compared with the control. In response to temperature and insecticide exposure, several metabolites and altered pathways were identified. Citrate, maltose, lipids, Nicotinate, Choline, Pyruvate and β-hydroxybutyrate were found as important components of major biological pathways such as tri-carboxylic acid cycle, branched amino acid degradation, glycolysis/gluconeogenesis, amino acid metabolism, lipid and carbohydrate metabolism, nucleotide PRPP pathway, and phospholipid metabolism. Furthermore, the results also suggest that the changes imposed by exposure to temperature and insecticides individually, are reversed with combined exposure, thus negating the impact of each other and posing a threat to the control of Aedes-borne diseases such as dengue, chikungunya, Zika and yellow fever.

## Introduction

Insect exposure to adverse environmental conditions is an inevitable day-to-day phenomenon^1^. With the consistent and fluctuating patterns of climatic conditions^2^, the insect vectors have to cope with the adverse situations. Studies have shown that insects, particularly mosquitoes have learned to adapt themselves up to a certain level^3,4^.

Along with adaptation to adverse climatic conditions, mosquitoes also face exposure to insecticides used by public health interventions for their control. Earlier studies have shown the susceptibility status of *A. aegypti* to insecticides^5^, but in recent years, resistance has been reported against major classes of insecticides from different parts of the country^6^ as well as globally^7^. Biologically, two major mechanisms play a role in the development of insecticide resistance, i.e. target site and metabolic resistance. Organochlorine (DDT) affect sodium ion channels, organophosphate (malathion) attach to the acetylcholinesterase enzyme^8^ at nerve cell ending and pyrethroids primarily target sodium ion channels for their neurotoxic actions along with ligand-gated ion channels^9^. Regards the link between temperature and insecticides, when mosquitoes are exposed to one stress (thermal), they develop cross-tolerance to another stress^10^. Glunt et al.^11^ have shown that a very likely possible range of daily temperature exposure leads to a significantly varying level of insecticide resistance in mosquitoes and insecticidal effectiveness is reported to be dependent on relevant temperature exposure^12^. Such studies necessitate the need to understand the underlying biological phenomena, as insecticide efficacy is affected by the interaction of environmental factors such as temperature.

Metabolomics generates the information about what is happening in a biological system, while genomics, transcriptomics, and proteomics are helpful in identifying what is expected to happen. Thus, metabolomics links the gap between genomics and phenomics^13^. Metabolomic research studies have been carried out on dengue virus transmission^14^, replication^15^, and diapause^16^ in dengue vectors, but the impact of temperature and insecticide on the metabolism remains unanswered. The metabolomics approach to study complex biological responses at the metabolic level offers the identification of multiple pathways of physiological responses and how they are connected to each other in response to external exposures^17–20^. Metabolites are the endpoint of every biological process, and changes in their concentration provide a clear picture of the impact of external exposure. Nuclear magnetic resonance (NMR) spectroscopy is one of the approaches to understand and measure the level of changes in metabolite concentration as a physiological response. It is expected that the physiological metabolic disturbance generated by exposure to temperature might exacerbate the insecticidal exposure.

The rationale behind the present study was the identification of common metabolic pathways affected by exposure to temperature and insecticides affecting the survival^21^ of mosquitoes. Therefore, it was thought prudent to understand the metabolomics of *A. aegypti* in response to exposure to different insecticides with and without temperature exposure.

## Results

**Figure 1:**
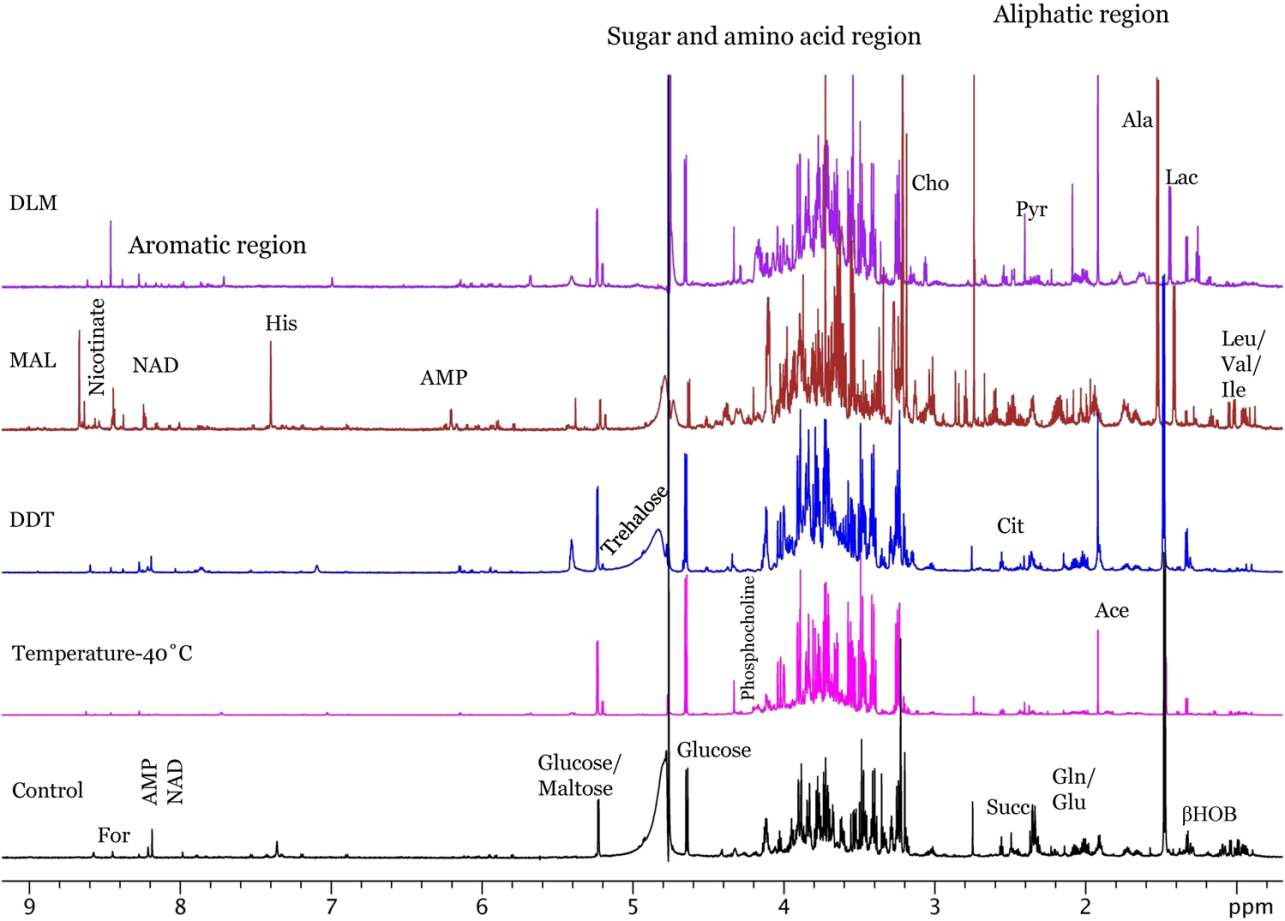
Stacked ^1^H-NMR spectra of *A. aegypti* in the control and one-hour of exposure to temperature-40 °C, DDT, MAL and DLM. Spectra were acquired at 700 MHz with a pre-sat pulse sequence of 128 scans at 25 °C and the sample pH was 7.4.

**Figure 2:**
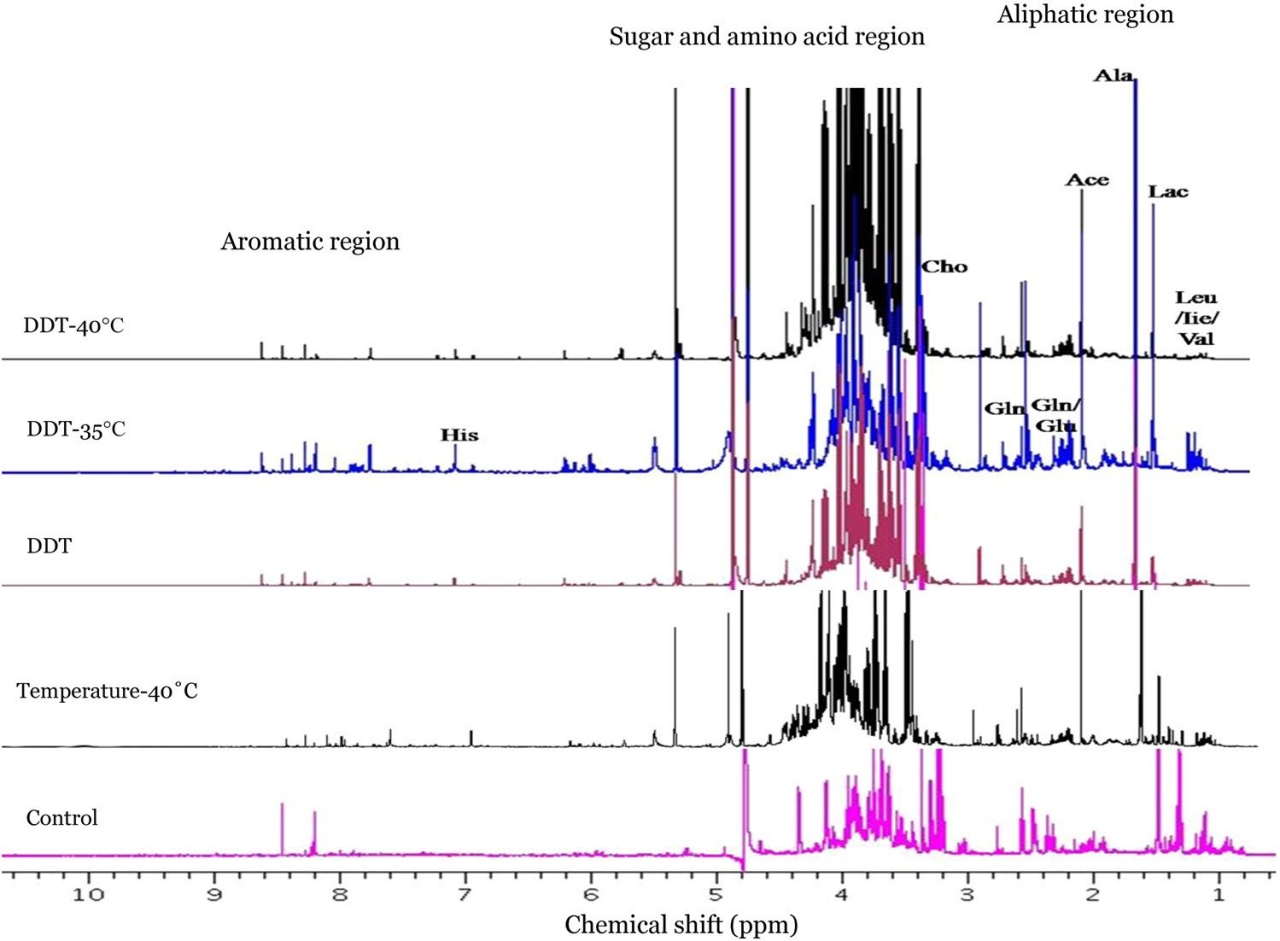
Stacked ^1^H-NMR spectra of *A. aegypti* exposed for one-hour to temperature (35 °C and 40 °C) followed by DDT and control groups (unexposed and only temperature exposure at 40 °C).

**Figure 3:**
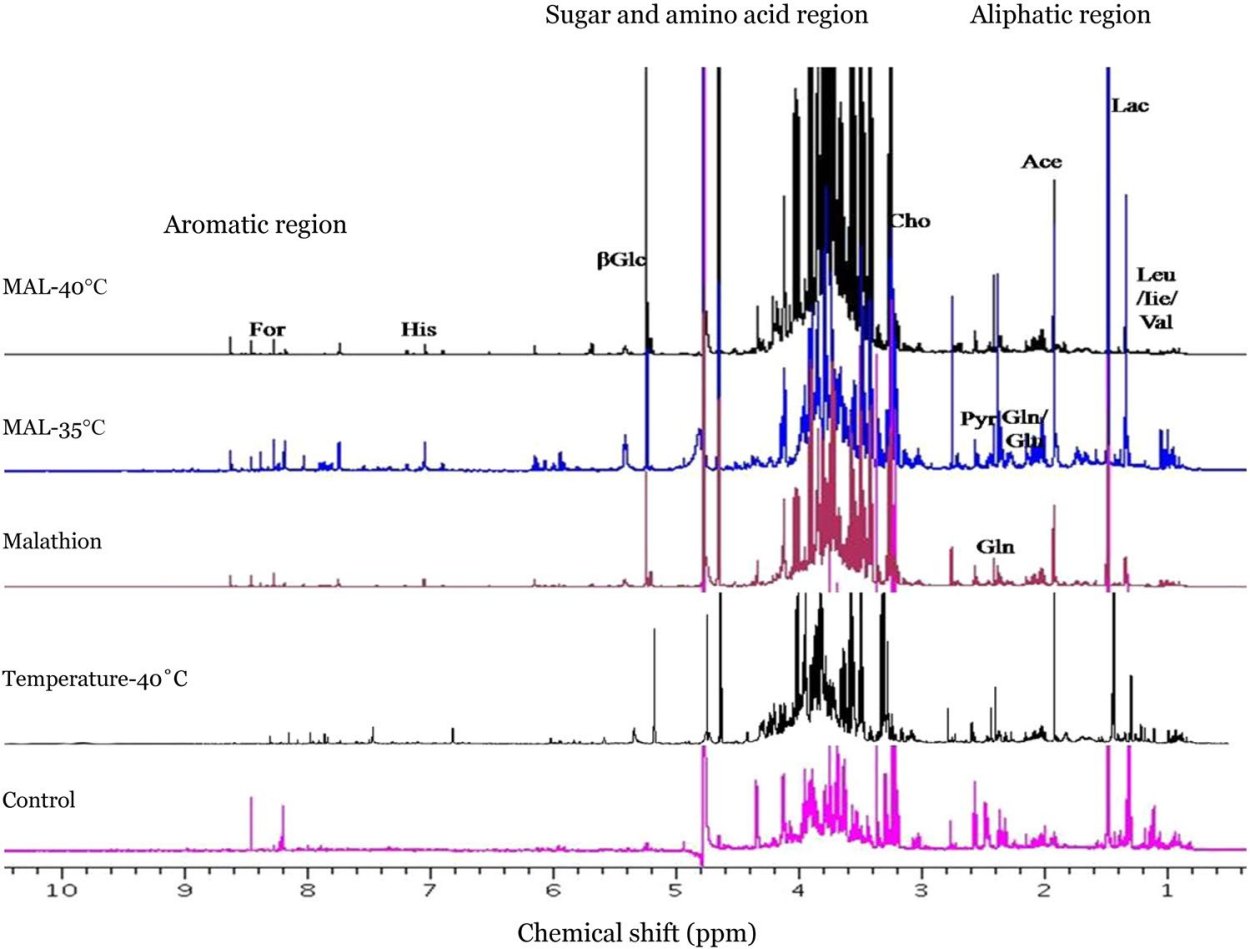
Stacked ^1^H-NMR spectra of *A. aegypti* exposed for one-hour to temperature (35 °C and 40 °C) followed by MAL and control groups (unexposed and only temperature exposure at 40 °C).

**Figure 4:**
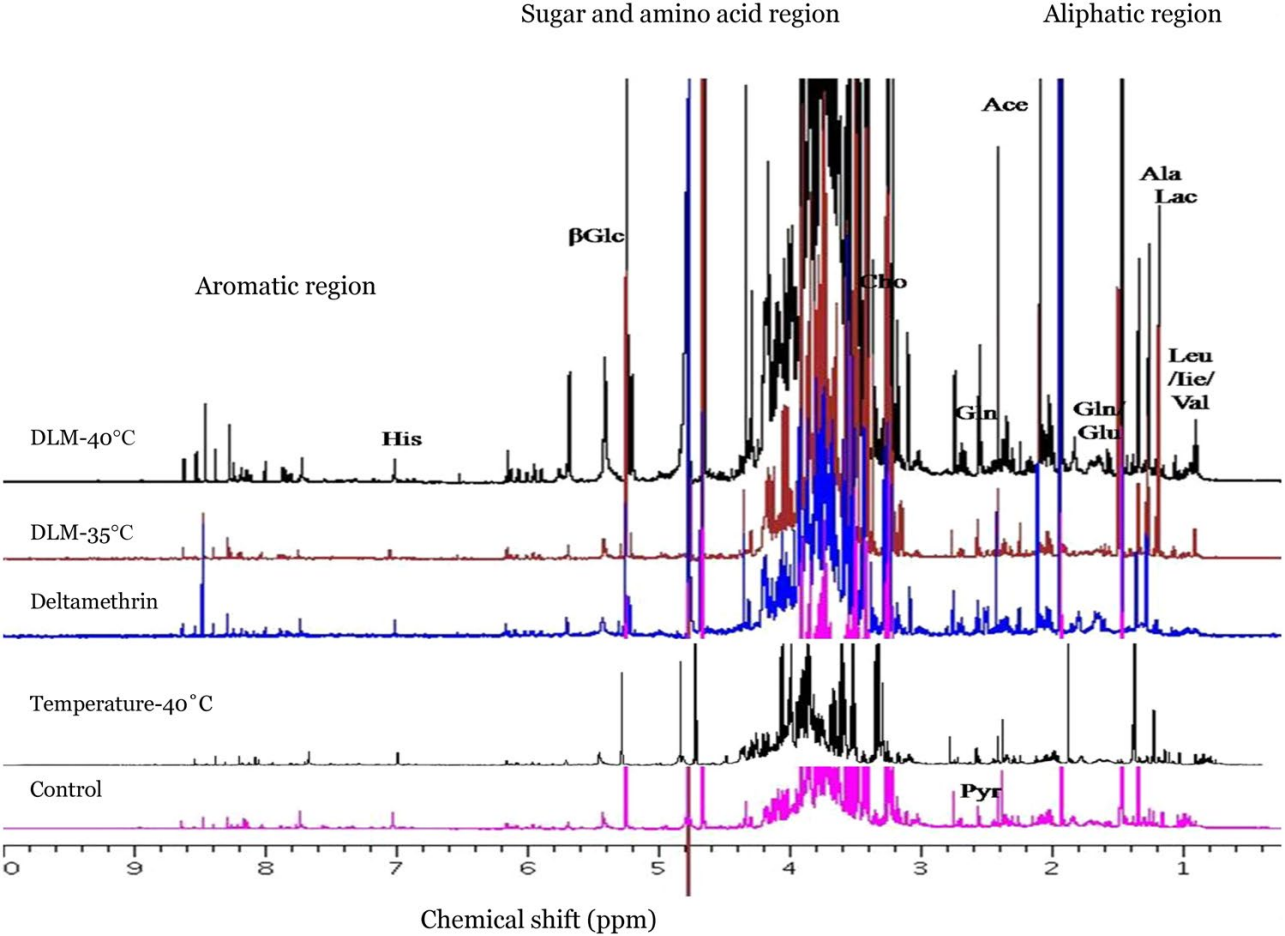
Stacked ^1^H-NMR spectra of *A. aegypti* exposed for one-hour to temperature (35 °C and 40 °C) followed by DLM and control groups (unexposed and only temperature exposure at 40 °C).

**Figure 5:**
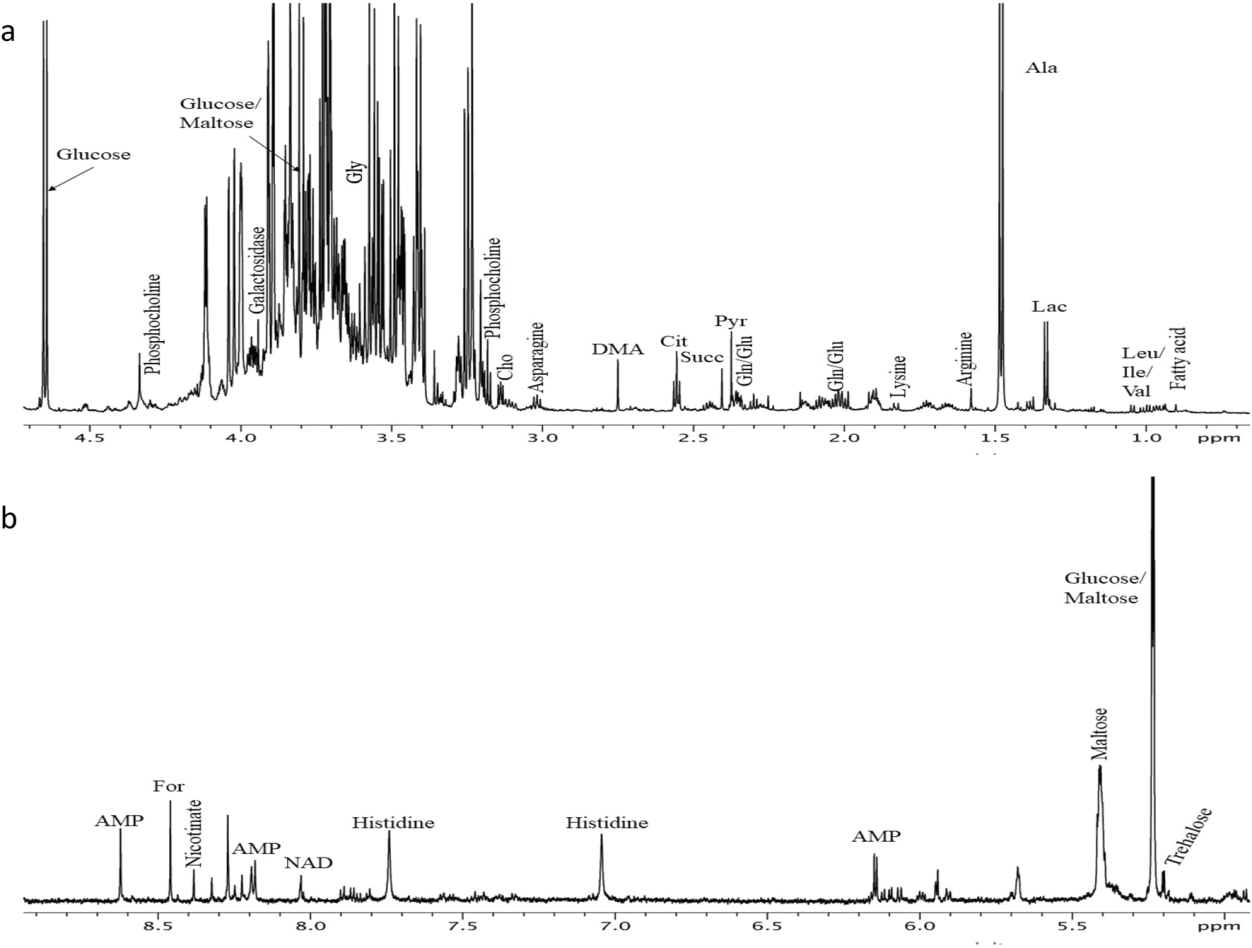
^**1**^H NMR spectrum of *A. aegypti* metabolites at 25 °C and pH 7.4. Well-resolved signals from the metabolites; (**a**) 0–5 ppm and, (**b)** 5–9 ppm are assigned.

In this paper, 1D ^1^H NMR spectra were obtained to study the change in metabolites following the temperature and insecticide exposure of *A. aegypti*. From Fig. 1, it is evident that exposure to temperature and/or insecticides (dichlorodiphenyl trichloroethane (DDT), malathion (MAL), and deltamethrin (DLM) resulted in a different metabolic profile as compared to control groups. Similarly, temperature exposure followed by insecticide treatment showed a clear picture of the impact of both the exposures (Figs. 2, 3, 4). A typical ^1^H NMR spectrum acquired from extracted samples of *A. aegypti* is shown in Fig. 5.

### Spectral assignment

Highly changing spectral regions were identified with significant differences between the exposures. From all different parts of the spectra, the major group of compounds were identified i.e. aliphatic region (0.8–2.8 ppm), sugar and amino acids (2.6–4.6 ppm) and aromatic (5.5–9.5 ppm) regions. The identified metabolites were: branched amino acids (leucine, isoleucine, valine), tricarboxylic acid cycle components (lactate, acetate, pyruvate, citrate, succinate), ketone bodies (β-hydroxybutyrate), amino acids (alanine, arginine, lysine, glycine, glutamate, glutamine, histidine, asparagine), amine (dimethylamine), lipids (choline), energy compounds (adenosine monophospahate; AMP), formate, fatty acids, sugars (maltose, glucose, galactoside, trehalose,), and nucleoside (uridine) (Fig. 5a,b).

### Univariate analysis

**Figure 6:**
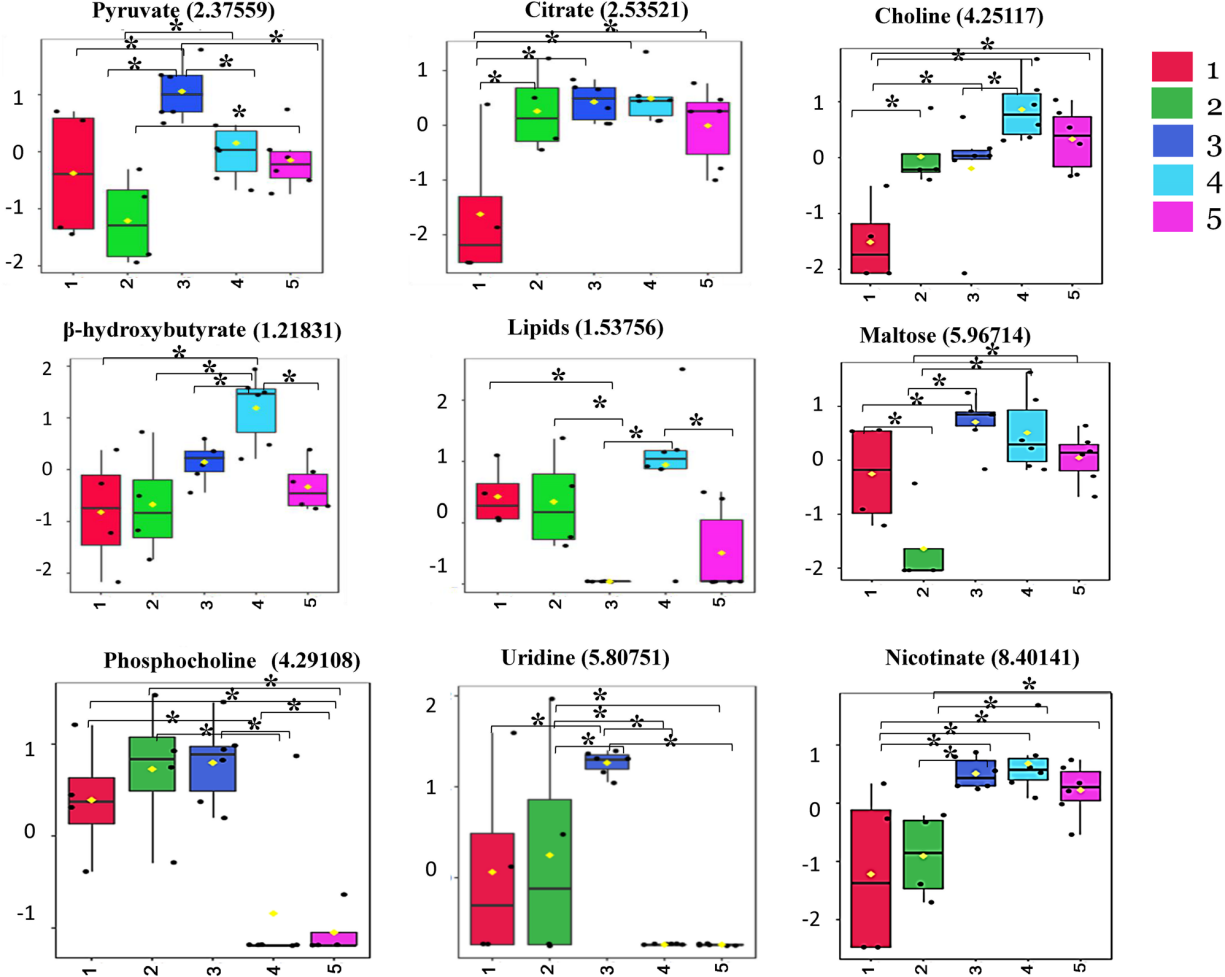
Box and Whisker plot for significantly altered pyruvate, citrate, β hydroxybutyrate, lipid, maltose, choline, phosphocholine, uridine and nicotinate of different exposures i.e. A-C*.* Asterisk (*) denote *p* < 0.05. Colored bar shows different exposure groups in *A. aegypti* (exposure A- 1-control; 2- temperature; 3- DDT; 4- MAL and 5- DLM, exposure B; 1-control; 2- temperature; 3- DDT; 4: DDT-35 °C and 5; DDT- 40 °C, and exposure C; 1-control; 2- temperature; 3- MAL; 4- MAL-35 °C and 5- MAL-40 °C).

Metabolomic data analysis carried out using, one-way ANOVA followed by post-hoc analysis, showed that seven metabolites namely; pyruvate, maltose, citrate, choline, b-hydroxybutyrate, lipids, nicotinate in exposure A (Table 1) while only one metabolite i.e. Uridine and phosphocholine in exposures B & C respectively, were significantly (*p* < 0.01) affected between the groups (Tables 2, 3). We did not identify any significantly affected metabolite under exposure D, i.e. DLM with varying temperature of 35 °C and 40 °C. Figure 6, shows the difference and significance of the metabolites.

**Table 1:** One-way ANOVA and significantly affected metabolites in lyophilized *A. aegypti* homogenate samples under exposure A.

Metabolites	Chemical shift (ppm)	f value	*p* value	−log10 (p)	FDR	Fisher's least significant difference* (LSD)
Maltose	5.96714	8.6815	0.00027	3.5728	0.03932	1 - 2; 3 - 1; 3 - 2; 4 - 2; 5 – 2**#**
Choline	4.25117	7.267	0.00077	3.1111	0.04591	2 - 1; 3 - 1; 4 - 1; 5 - 1; 4 – 3**#**
Nicotinate	8.40141	6.5536	0.00138	2.8601	0.04591	3 - 1; 4 - 1; 5 - 1; 3 - 2; 4 - 2; 5 – 2**#**
β Hydroxybutyrate	1.21831	6.4771	0.00147	2.8323	0.04591	4 - 1; 4 - 2; 4 - 3; 4 – 5**#**
Lipids	1.53756	6.1666	0.00191	2.7182	0.04591	1 - 3; 2 - 3; 4 - 3; 4 – 5**#**
Pyruvate	2.37559	6.0828	0.00206	2.687	0.04591	3 - 1; 3 - 2; 4 - 2; 5 - 2; 3 - 4; 3 – 5**#**
Citrate	2.53521	6.0119	0.00219	2.6604	0.04591	2 - 1; 3 - 1; 4 - 1; 5 – 1**#**

*Fisher's LSD: Fisher's least significant difference; a two-step testing procedure for pairwise comparisons of different exposure groups.

^**#**^Control = 1; Temperature-40 °C  = 2; DDT = 3; MAL = 4; DLM = 5.

**Table 2:** One-way ANOVA and significantly affected metabolites in lyophilized *A. aegypti* homogenate samples under exposure B.

Metabolites	Chemical shift (ppm)	f value	*p* value	−log10 (p)	FDR	Fisher's least significant difference (LSD)
Uridine	5.80751	9.864	0.00012	3.9268	0.01752	3 - 1; 3 - 2; 2 - 4; 2 - 5; 3 - 4; 3 - 5^$^

^**$**^Control = 1; Temperature-40 °C  = 2; DDT = 3; DDT-35 °C = 4; DDT-40 °C = 5.

**Table 3:** One-way ANOVA and significantly affected metabolites in lyophilized *A. aegypti* homogenate samples under exposure C.

Metabolites	Chemical shift (ppm)	f value	*p* value	−log10 (p)	FDR	Fisher's least significant difference (LSD)
Phosho-choline	4.29108	9.2708	0.00025	3.6035	0.03663	1 - 4; 1 - 5; 2 - 4; 2 - 5; 3 - 4; 3 – 5§

^§^Control = 1; Temperature-40 °C  = 2; MAL = 3; MAL-35 °C = 4; MAL-40 °C = 5.

### Multivariate analysis

**Figure 7:**
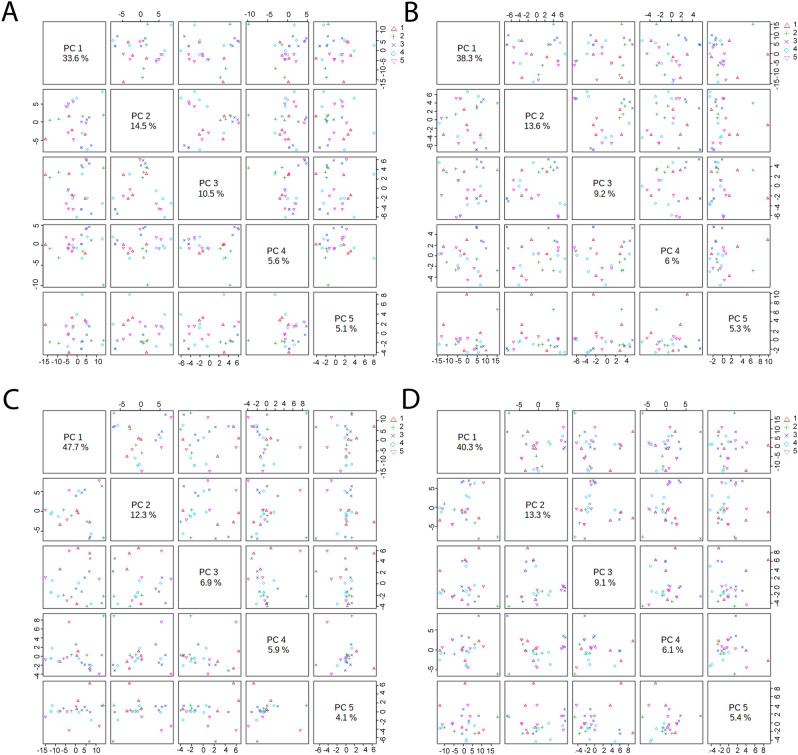
Principal component analysis: PC1 accounted for 33.6%, 38.3%, 47.7%, and 40.3% of variation, followed by PC2 i.e. 14.5%, 13.6%, 12.3%, and 13.3% in exposure (**A**), (**B**), (**C**) and (**D**), respectively in *A. aegypti*. The smallest part of the changes picked up by PC5 i.e. 5.1%, 5.3%, 4.1% and 5.4% in exposures (**A**), (**B**), (**C**) and (**D**), respectively. Exposure (**A**)- 1-control; 2- temperature; 3- DDT; 4- MAL and 5- DLM, exposure (**B**); 1-control; 2- temperature; 3- DDT; 4: DDT-35 °C and 5; DDT- 40 °C, exposure (**C**); 1-control; 2- temperature; 3- MAL; 4- MAL-35 °C and 5- MAL-40 °C and, exposure (**D**); 1-control; 2- temperature; 3- DLM; 4- DLM-35 °C and 5- DLM-40 °C.

Principal component analysis (PCA) was carried out to identify the changes in metabolic profile of *A. aegypti* exposed to A, B, C and D. The resultant PCA score plot for the discrimination values of exposures A-D is shown in Fig. 7. PC1 shows higher part of variance i.e. 33.6%, 38.3%, 47.7%, and 40.3% while in PC2 the variance was 14.5%, 13.6%, 12.3%, 13.3% in exposures-A, B, C, D respectively, suggesting high amplitude changes between the groups.

**Figure 8:**
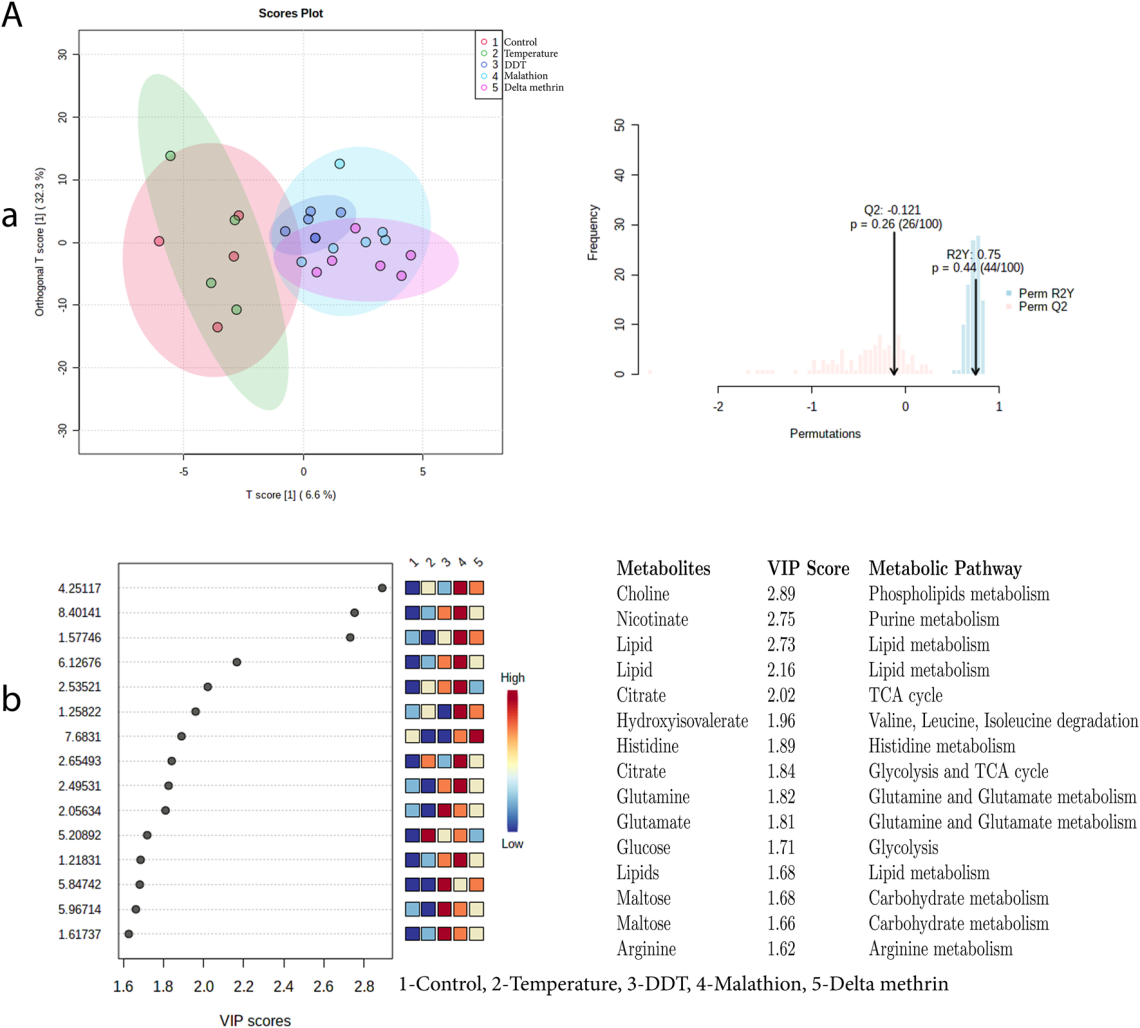
Exposure A: (**a**) denotes the supervised OPLS-DA score plot showing discrimination of metabolites and validation of OPLS-DA by permutation test performed with 100 random permutations while (**b**) denotes VIP score of identified metabolites (intensity given in low to high colour gradient) and associated metabolic pathways between exposed groups i.e. Control = 1; Temperature-40 °C = 2; DDT = 3; MAL = 4; DLM = 5.

Exposure A, The OPLS-DA score plot between the control and temperature and insecticide (DDT, MAL, DLM) exposure showed clear discrimination and the results were validated by permutation tests performed with 100 random permutations, resulting in R2 = 0.75 and Q2 = − 0.121 (*p* > 0.05). OPLS-DA score plot analysis (Fig. 8), further identified 15 discriminating metabolites, of which two (glucose and citrate) were upregulated while the other 12 downregulated in the temperature exposed group (Fig. 8). In the DDT exposed group, seven metabolites (nicotinate, lipid, citrate, glutamine, glutamate, maltose and arginine), were upregulated and four metabolites (histidine, hydroxyvalerate, choline and citrate) were downregulated. Except for maltose, the remaining 13 metabolites were upregulated (choline, nicotinate, lipid, citrate, hydroxyvalerate, histidine, citrate, glutamine, glutamate, glucose, lipids, maltose, arginine) in MAL exposure. In DLM exposure, five metabolites (choline, lipid, histidine, hydroxyvalerate, maltose) were upregulated and two metabolites (citrate, glucose) were downregulated.

**Figure 9:**
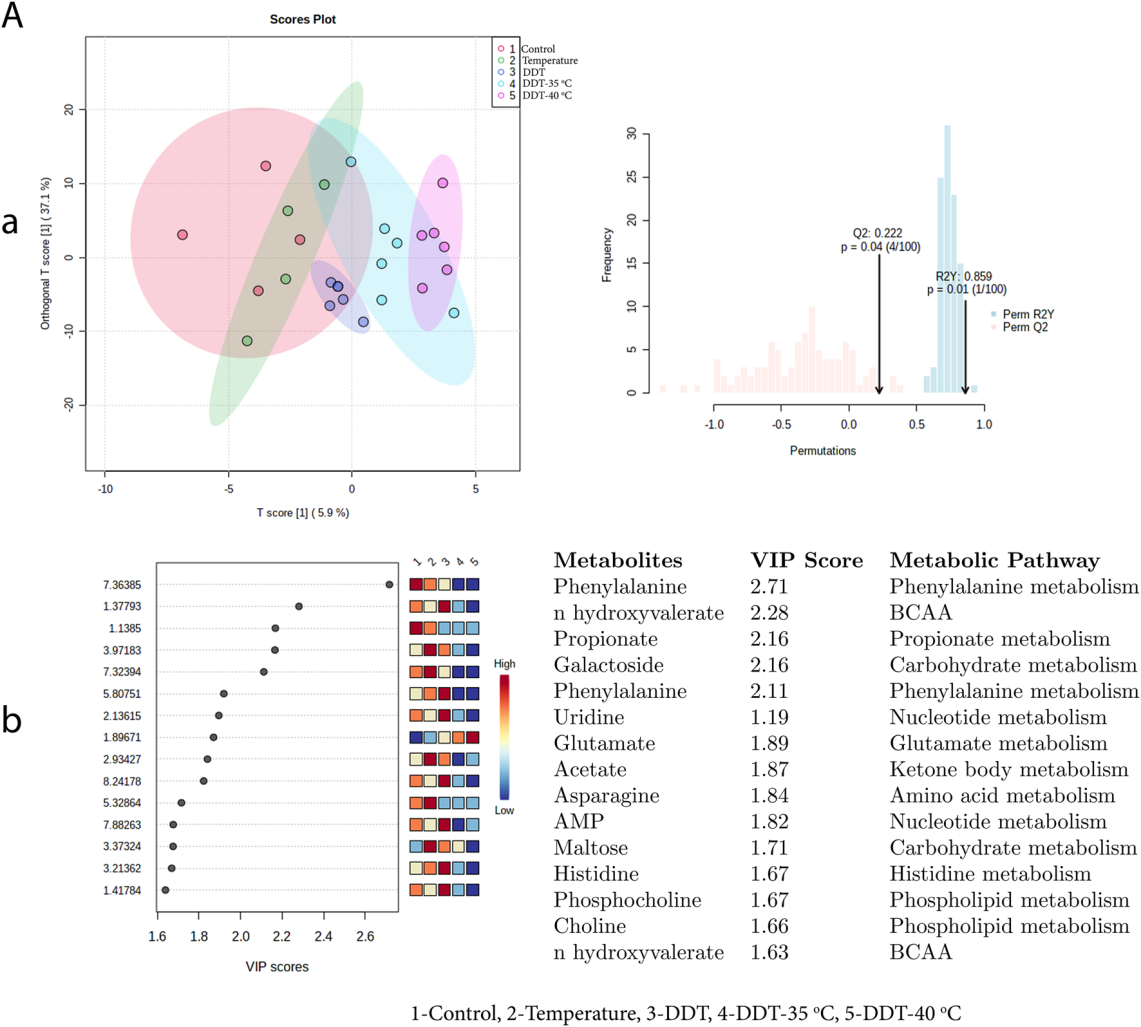
Exposure B: (**a**) denotes the supervised OPLS-DA score plot showing discrimination of metabolites and validation of OPLS-DA by permutation test performed with 100 random permutations while (**b**) denotes VIP score of identified metabolites (intensity given in low to high colour gradient) and associated metabolic pathways between exposed groups i.e. Control = 1; Temperature-40 °C = 2; DDT = 3; DDT-35 °C = 4; DDT-40 °C = 5.

**Figure 10:**
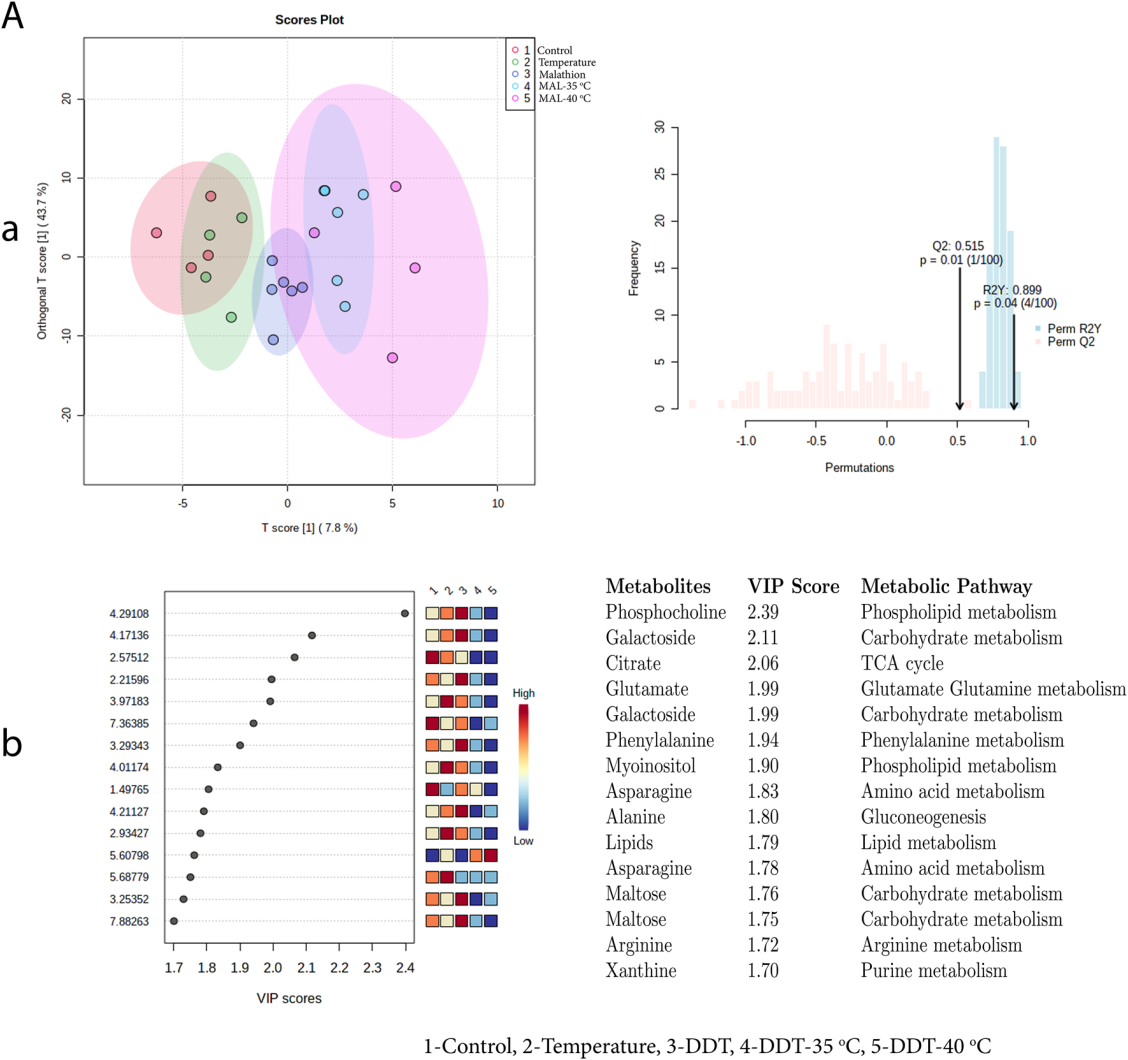
Exposure C: (**a**) denotes the supervised OPLS-DA score plot showing discrimination of metabolites and validation of OPLS-DA by permutation test performed with 100 random permutations while (**b**) denotes VIP score of identified metabolites (intensity given in low to high colour gradient) and associated metabolic pathways between exposed groups i.e. Control = 1; Temperature-40 °C = 2; MAL = 3; MAL-35 °C = 4; MAL-40 °C = 5.

**Figure 11:**
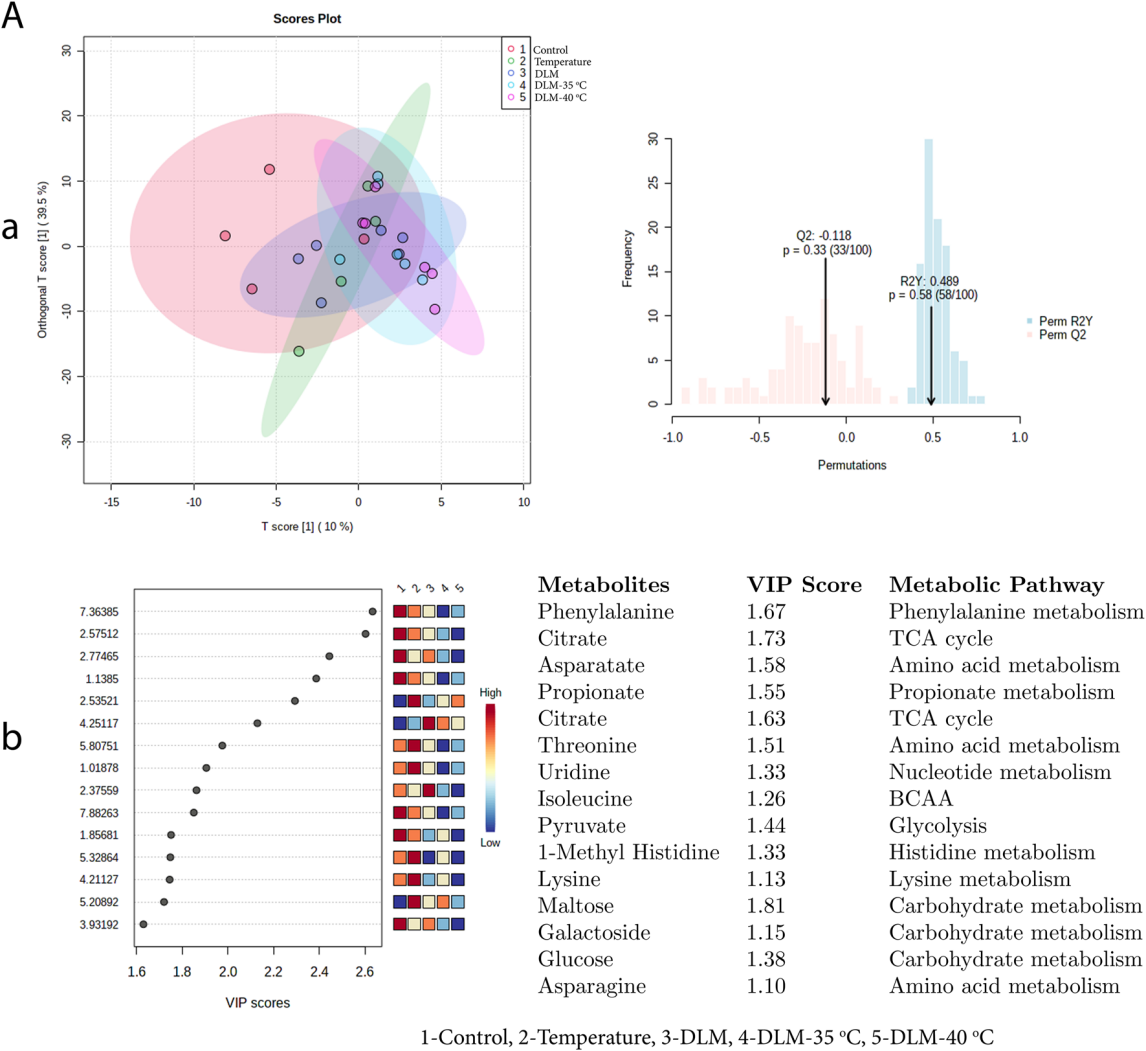
Exposure D: (**a**) denotes the supervised OPLS-DA score plot showing discrimination of metabolites and validation of OPLS-DA by permutation test performed with 100 random permutations while (**b**) denotes VIP score of identified metabolites (intensity given in low to high colour gradient) and associated metabolic pathways between exposed groups i.e. Control = 1; Temperature-40 °C = 2; DLM = 3; DLM -35 °C = 4; DLM -40 °C = 5.

OPLS-DA score plot (Figs. 9, 10) for exposure B and C i.e. DDT and MAL at 35 °C and 40 °C temperature, showed clear discrimination from the control. The results were validated by permutation test performed with 100 random permutations, resulting in R2 = 0.85 and Q2 = 0.22 (*p* < 0.05) (exposure B) and R2 = 0.89 and Q2 = 0.51 (*p* < 0.05) (exposure C), which suggested robustness of the model. However, the OPLS-DA score plot did not show significant discrimination between Control and DLM exposure at 35 and 40 °C temperature (exposure D) (R2 = 0.48, Q2 = − 0.11, *p* > 0.05) (Fig. 11).

Furthermore, by using variable importance in projection (VIP) scores (a VIP score of > 1.0 is considered statistically significant*)* with PLSDA analysis, 15 discriminating metabolites were found; of which except citrate and the remaining 14 metabolites were found to be downregulated, particularly in temperature along with DDT exposure (B). On the other hand, variable importance in projection (VIP > 1.0) scores during the exposure C and D OPLS–DA analysis showed that except maltose in MAL and citrate at 40 °C and choline and glucose at 35 °C in DLM exposure, other metabolites were downregulated (Fig. 11b). VIP score plot analysis revealed that in all three exposures i.e. B-D, metabolites that were highly discriminated during individual exposure to temperature and insecticides, became less discriminated during the combined exposure to temperature followed by the insecticide.

### Heat map analysis

**Figure 12:**
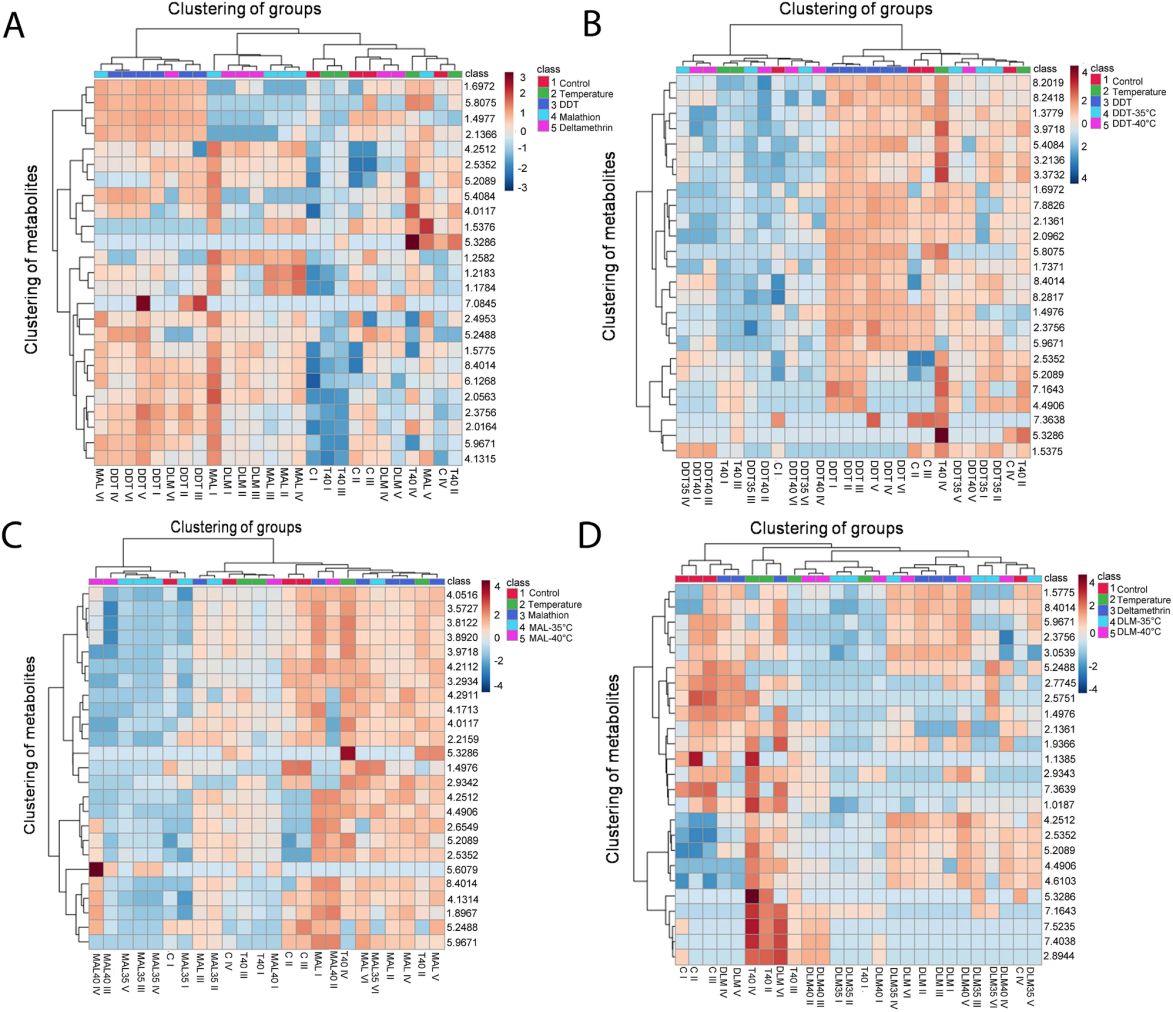
Heat map analysis of identified differential metabolites from NMR based metabolomics. Each row represents the ion intensity of a metabolite after rescaling the data (mean centering and unit variance). Each column shows the metabolic patterns of different exposure classes in *A. aegypti* (exposure (**A**)- 1-control; 2- temperature; 3- DDT; 4- MAL and 5- DLM, exposure (**B**); 1-control; 2- temperature; 3- DDT; 4: DDT-35 °C and 5; DDT- 40 °C, exposure (**C**); 1-control; 2- temperature; 3- MAL; 4- MAL-35 °C and 5- MAL-40 °C and, exposure (**D**); 1-control; 2- temperature; 3- DLM; 4- DLM-35 °C and 5- DLM-40 °C). The red color of the tile indicates high abundance and green indicates low abundance of metabolites.

Heat map visualization was performed using the normalized concentration of 25 metabolites under different exposure experiments i.e. A-D in *A aegypti* is shown in Fig. 12. A dendrogram produced by performing hierarchical clustering, revealed that clustering of metabolites in DDT exposure is dissimilar from other four groups in exposure A. Cluster metabolites were positively correlated in DDT and MAL but negatively correlated in the control and temperature groups (Fig. 12A). During exposure B & C, clustering of metabolites was found to be negatively correlated between insecticides i.e. class 3 and temperature exposure followed by insecticides i.e. class 4 & 5 (Fig. 12B,C). In exposure D, metabolite clustering was found in combined exposure classes i.e. 4 & 5 and negatively correlated with control groups i.e. class 1, 2, and 3 (Fig. 12D).

## Discussion

Our study has elicited an understanding of metabolic changes due to temperature and insecticide exposure in *A. aegypti*. The most affected metabolites found in the present study are components of major biological pathways namely, tri-carboxylic acid cycle, branched amino acid degradation, glycolysis/ gluconeogenesis, amino acid metabolism, lipid and carbohydrate metabolism, nucleotide PRPP pathway, and phospholipid metabolism. Furthermore, the results also show that exposure to temperature and insecticides combinedly increases the overall rate of knockdown in *A. aegypti*, which is resistant to DDT, malathion and pyrethroids^22^.

Out of nine identified metabolites, seven were found to be significantly affected in exposure A, i.e. exposure to individual temperature and insecticides. Of the seven metabolites, pyruvate, maltose, citrate, nicotinate and β- hydroxybutyrate are part of energy generating pathways, i.e. glycolysis/ gluconeogenesis, tri-carboxylic acid cycle^23^ during biotic/ abiotic stress^24^ and flying^25^ etc.

Significant changes in the concentration of pyruvate, a product of glycolysis and a precursor for gluconeogenesis, reflect that individual exposure to temperature and insecticides increases the energy needed to withstand the exposure induced stress in the vector. Studies have shown that pyruvate metabolism through glycolysis/ gluconeogenesis is a key part of oxidation stress upon viral infection in *A. aegypti*^26^ and through oxidative phosphorylation in the mitochondria of flight muscles of insects during flight^27,28^. Furthermore, pyruvate has also been found to be responsible for the highest respiratory rate^29^ and to function as a substrate for alanine aminotransferase during effective nitrogen waste disposal in *A. aegypti*^30,31^. Along with pyruvate, maltose another energy metabolite, was found to be significantly altered after the aforementioned exposures which may be due to dietary supply to the mosquitoes in the form of raisin. Biochemical maltose, may be converted to glucose or trehalose (KEGG pathway analysis). In this study, after exposure to temperature, the glucose level increased, which may be an outcome of hindrance in related metabolic activities due to heat stress (Fig. 8b). Similarly, the presence of an insignificant amount of trehalose is evident from the spectra suggesting that trehalose was produced but simultaneously utilized by the mosquito vector during the exposure, as it helps combat abiotic stresses^32^ and acts as a source of energy for flight and development in insects^33,34^. The results of exposure A show a significant impact on citrate, one of the central molecules of the tricarboxylic acid cycle, which further suggests that exposure hampers the ultimate event of energy production. The metabolic profile of the salivary gland has shown that pyruvate dehydrogenase, and isocitrate dehydrogenase (the enzymes of the citric cycle) are upregulated while citrate synthase, malate dehydrogenase, and succinyl CoA-synthase are downregulated with DENV-2 infection^35^. Two other significant metabolites, nicotinate and β-hydroxybutyrate, also reflect the need for energy to withstand the temperature and insecticide imposed stress in the vector. As KEGG pathway analysis of *Aedes* shows that nicotinate is important for NAD + biosynthesis while β-hydroxybutyrate has been identified as a source of energy in other insects^36^. Furthermore, oxidation of ketone bodies is greatly reduced under starving and stress conditions^37^.

The remaining two metabolites under exposure A i.e. Choline and lipids are associated with insecticide susceptibility/resistance^38^ and thermal tolerance^39^ in mosquitoes. The majority of insecticides function as inhibitors of acetyl cholinesterase enzymes, which hinder the function of acetylcholine at synapses^40^. Acetylcholine synthesis takes place from choline and acetyl coenzyme-A in the presence of choline acetyltransferase at the synaptic cleft. In the light of the available literature and present evidence, the impact of malathion^41^ on choline in targeting acetylcholine esterase is expected but with respect to exposure to temperature and other insecticides^42^, which needs to be elucidated. While studying the cuticular resistance mechanism, Balabanidouet al.^43^ reported that lipid biosynthesis significantly increased in resistant mosquitoes compared with susceptible. Apart from this, the importance of lipid metabolism has been reported in the from ovary, midgut, exoskeleton and flight muscle^44^ of the insects. Corroborating previous findings, our results suggest that lipids play an important role in the development of thermal resistance and protection against exposure driven desiccation^45,46^ and stress^47,48^.

Apart from the statistically significant metabolites, the temperature exposure shows a reduction in the amount of glutamate/ glutamine as compared to control, which highlights the importance of glutamine during heat stress^49^. Amino acids are known to maintain equilibrium in cells under nonstress conditions, and disturbances in their concentration show a common impact of exposures. The spectral presence of hydroxyvalerate shows the degradation of nonpolar branched chain amino acids, i.e., leucine, valine and Isoleucine, which are normally found in every tissue. Although we did not find a significant amount of branched amino acids, earlier studies undertaken on *A. aegypti* have suggested the role of the branched chain amino acid degradation pathway in maintaining the bacterial load in the midgut during viral infection in vectors and for further transmission^50^.

The part B of exposure i.e. Combined exposure to temperature and DDT showed significant changes in uridine metabolite. Uridine diphosphate (UDP)-glycosyltransferases have been extensively reported in insect antennae^51,52^ and a differential expression has also been reported from resistant strains against DDT^53^ and pyrethroids^54^. DDT is widely used insecticide, and the majority of insects, including *A. aegypti* have become resistant to it^21^. It has been reported that hort –term exposure of DDT-resistant *Anopheles arabiensis* to 37 °C and 39 °C temperatures increases the resistance against pyrethroid^55^. Such studies are desired to ascertain the role of temperature exposure in the insecticide resistant *Aedes.*

The combined exposure to temperature and malathion (exposure C) affects only phosphocholine, a major component of the polar head group of plasma membrane phospholipids^56^. As per the well-studied mechanism, malathion exposure prevents breakdown of acetylcholine^57^ while the results of our study suggest that with exposure to temperature followed by malathion, it may affect the cellular behavior by affecting phospholipid composition in mosquitoes. Altered phospholipid pathways have also been reported in live *Mycobacterium ulcerans* infected mosquitoes^58^. In the absence of supporting data, it needs further elucidation of the results is needed, as combined exposure also increases the rate of knockdown at 35 °C and decreases at 40 °C.

Exposure D did not bring out any significant result in terms of metabolites warranting strengthening data in the light of developed pyrethroid resistance in *A. aegypti*. One study had shown that deltamethrin resistant mosquitoes develop thick cuticle layer in comparison to susceptible ones^59^.

Significant discrimination of seven metabolites under exposure A, and only one or nil in other sets of exposure suggests that temperature exposure affects insecticide efficacy in *A. aegypti*^60^. Further, the exposure to temperature helps in developing or increasing the cross-resistance towards insecticides^10^. *A. aegypti* has been reported robust in respect to thermal tolerance as well as has already developed resistance towards insecticides. The findings of the present study indicating exacerbation of insecticide resistance with increased temperature (which is expected due to climate change), poses threat on vector control in hot climate zones. Further studies on biological assays are required to compliment the results of present study.

## Conclusion

This study used a metabolomics profile approach to identify different metabolic signatures related to temperature and insecticidal exposure in *A. aegypti*. The results demonstrated that tri-carboxylic acid cycle, branched amino acid degradation, glycolysis/ gluconeogenesis, amino acids, lipid, carbohydrate and phospholipid metabolism were significantly affected. Under exposure A, seven metabolites were found, while in category B, uridine, category C, phosphocholine and no significant metabolite were found in category D. The changes imposed by exposure to temperature and insecticides individually were reversed with combined exposure, thus negating the impact of each other. Based on present finding, it is expected that temperature may affect the metabolic pathways of the insecticides being used in hot climatic conditions. Furthermore, the findings of the present study strengthen the hypothesis that the metabolomics approach is a very good tool for understanding the physiological responses to the temperature and insecticide exposure.

## Methods

### Adult vector

**Figure 13:**
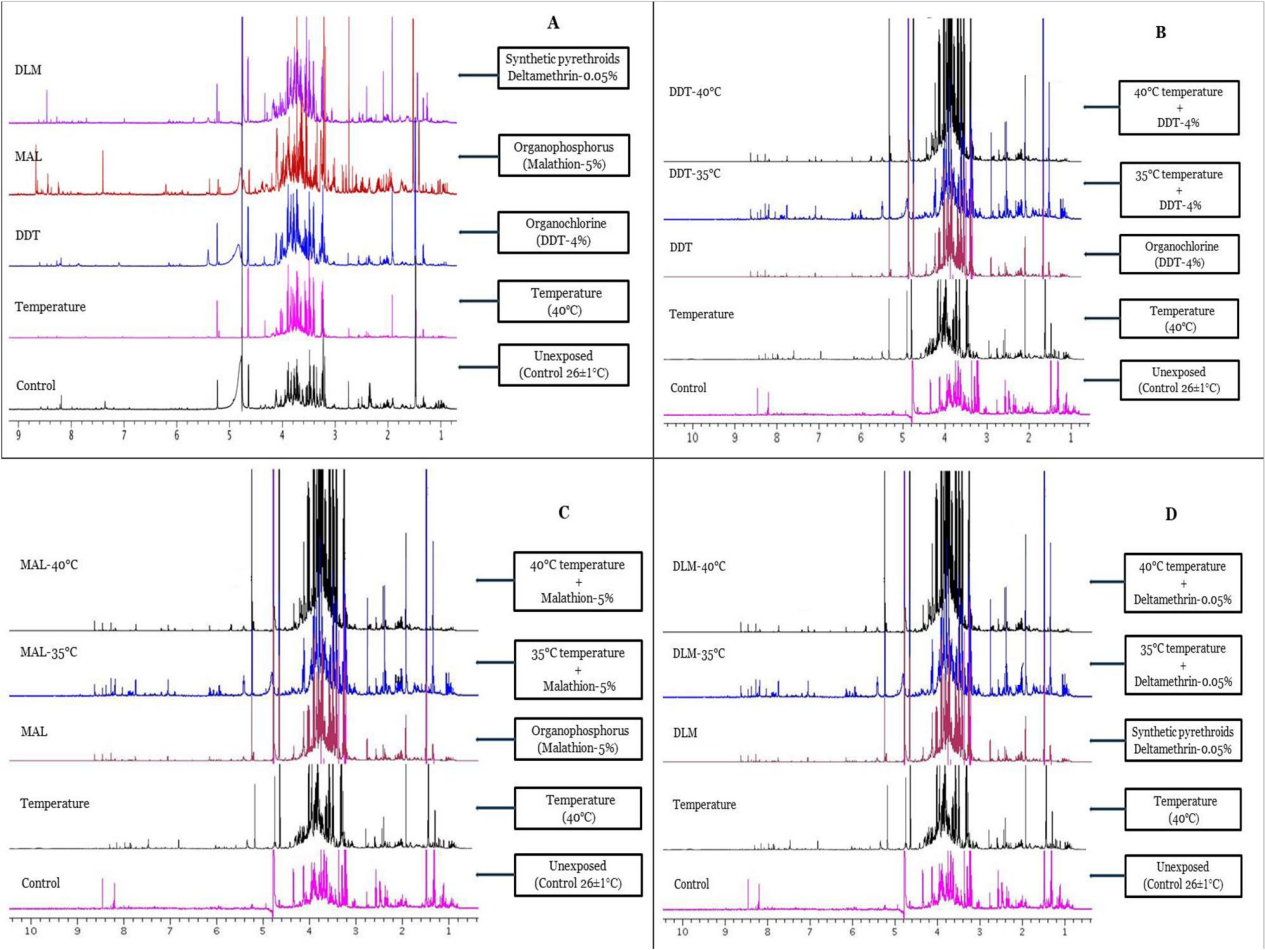
Study flow chart; (**A**)- 1-Hour exposure of *A. aegypti’* at 40 °C temperature and 1-Hour and insecticides i.e. DDT- 4%, malathion-5% and deltamethrin- 0.05% and, (**B–D**) are 1-Hour exposure of *Aedes aegypti’* at 35 °C and 40 °C temperature followed by 1-h exposure to the insecticides i.e. DDT- 4%, malathion-5% and deltamethrin- 0.05%.

*Aedes* (Stegomyia) *aegypti* (Linnaeus, 1762), used in the study, was taken from the insectary at ICMR-National Institute of Malaria Research, Delhi. To generate the metabolic profile of the impact of temperature and insecticides, 2 to 3 day old female mosquitoes (six replicates of 50 mosquitoes each) housed in Barraud’s cages were exposed to temperature in a Percival Incubator (USA). The insecticides used for exposure were DDT (4%), malathion (5%) and deltamethrin (0.05%). The insecticide-impregnated papers were procured from the University Sans Malasia, Malaysia). The insecticide susceptibility test kit of the WHO was used for one-hour exposure (Fig. 13). No supplementary food was given to mosquitoes during the exposure time. The control replicates were housed at optimum insectary temperature conditions of 26 °C ± 1 °C and 70% ± 1.5% RH. As mosquitoes were given one-hour exposure to the insecticides and immediately frozen for further sample preparation, rather than mortality percentage, the knockdown percentage for every exposure was also calculated (supplementary table s1).

The metabolic profiles were generated as per the work flow chart (Fig. 13A). Exposure A: one-hour exposure of *A. aegypti’* to 40 °C temperature and to insecticides i.e. DDT- 4%, malathion-5% and deltamethrin- 0.05% (Fig. 13A). While B, C, and D (Fig. 13B–D) involves one-hour exposure of *Aedes aegypti’* to 35 °C and 40 °C temperatures followed by one-hour exposure to the insecticides, i.e. 1-DDT-4%, 2-malathion-5% and 3-deltamethrin- 0.05%.

### Sample preparation

After exposure, the mosquitoes were immediately snap-frozen in the liquid nitrogen to arrest the metabolic reactions. The frozen tissue was weighed, crushed and thoroughly homogenized in 6% perchloric Acid. The homogenate was then centrifuged at 10,000 rpm for 10 min, after which the supernatant was collected and neutralized using 3 M potassium hydroxide (KOH) at pH 7.0. The obtained supernatant was then subjected to 8–10 h of lyophilization. After lyophilization, the sample powder was dissolved in 760 μl of deuterium oxide (D_2_O), and 40 μl (0.5 mM) sodium trimethyl-silyl-[2, 2, 3, 3-H4] propionate (TSP) was added to the sample which served as a chemical shift reference for the proton (^1^H) NMR spectroscopy. Approximately 600 μl of total sample, volume (800 μl) was transferred into a 5 mm NMR tube.

### ^1^H NMR spectroscopy

#### NMR parameters

Proton NMR spectra of the samples were obtained using a 700 MHz spectrometer (Agilent, USA). One dimensional (1D) ^1^H NMR spectra of samples were acquired with a single 90° pulse with water suppression (using a presaturation RF pulse). A relaxation delay of 5 s was used for the experiment. A total of 128 scans were collected with 32 K data points over a spectral width of 9124.1 Hz. Two dimensional (2D) total correlation spectroscopy (TOCSY) experiments were carried out for assignments of resonances (twodimensional (2D; zTOCSY): 2 K data point, relaxation delay = 2.5 s; number of increments = 400 (t1); number of scans = 16; spin lock = 80 ms).

#### NMR spectra processing

NMR spectra were processed for metabolomics analysis which included scaling, alignment, exclusion of selected signals arising from solvent and TSP, binning and transformation. The data were processed using the Vnmrj 2.3A software (Agilent Technologies). The NMR spectra were manually phased and adjusted, and the baseline was corrected using MestReNova software (TSP chemical shift at 0.00 ppm). The complexity of spectral data was reduced before statistical analysis by using spectral binning and a data set with manageable proportion was produced. In our study, the spectral regions at 0.5– 12 ppm were segmented into bins with equal widths of 0.04 ppm. The spectral region 4.94–4.66 ppm (residual water signal) was excluded. The identification of selected metabolites was crosschecked from the Spectral Data Base (SDBS) and published literature.

### Statistical analysis

Statistical analysis was carried out using SPSS software (SPSS Inc. Chicago, IL, USA) and MetaboAnalyst4.0 software (www.metaboanalyst.ca). For comparison between two groups, t-test was used. A *p* value of < 0.05 was considered significant. To analyze the data obtained in the studies, univariate (ANOVA) and multivariate i.e. Principal component analysis or PCA, orthogonal partial least squares discriminant analysis OPLS-DA, variable importance to projection (VIP) score, and statistical approaches were applied to the samples (a VIP score of > 1.0 was considered to be statistically significant). PCA is one such method that has been used extensively in metabolomics. PCA, PLS-DA, and OPLS-DA were applied to the metabolomic profile dataset to see the separations between the groups. Statistical models from PLS-DA were validated by random permutation of the response variable and comparison of the goodness of fit (R2) and predictive ability (Q2) values. Finally, hierarchical cluster analysis by k-means of the groups and Pearson’s correlation was used to generate the heat maps for identification of the clustering pattern. A dendrogram was produced using the normalized concentration of 25 metabolites for clustering of sample groups in the control and exposed groups. In the dendrogram, shorter the lines indicate a closer relationship, while lengthier the vertical lines indicate less the similarity between the exposure groups.

## Supplementary Information

Supplementary Information.

## Data Availability

The analyzed results data are available with the authors, available on request.
