# Supplementary material for: Untargeted metabolomics-based response analysis of temperature and insecticide exposure in Aedes aegypti
Source: Sci Rep. 2022 Feb 8;12:2066. doi: 10.1038/s41598-022-05630-z (PMC8825845; doi:10.1038/s41598-022-05630-z)
Supplement: Supplementary file 1 — Supplementary Information. [file 41598_2022_5630_MOESM1_ESM.docx]

Supplementary table s1:

| Exposure  (1 hour each) | DDT (4%) | | Malathion (5%) |  | Deltamethrin (0.05%) | |
| --- | --- | --- | --- | --- | --- | --- |
|  | Mosquito tested (n) | Knock down % (1hour) | Mosquito tested (n) | Knock down % (1hour) | Mosquito tested (n) | Knock down % (1hour) |
| Control | 120 | 0 | 240 | 95 | 240 | 88 |
| 35°C + Insecticide | 210 | 15 | 210 | 72 | 240 | 91 |
| 45°C + Insecticide | 255 | 9 | 240 | 81 | 270 | 99 |

Table s1: Knock down percentage of insecticides exposed Aedes aegypti via discriminating concentration bioassays
